# The Effect of Additive and Multiplicative Cyclic Perturbations on Noise-Induced Tipping Dynamics

**DOI:** 10.3390/e27121255

**Published:** 2025-12-13

**Authors:** Igor A. Khovanov, Natasha A. Khovanova

**Affiliations:** School of Engineering, University of Warwick, Coventry CV4 7AL, UK; n.khovanova@warwick.ac.uk

**Keywords:** tipping point, fold bifurcation, noise-induced transition, periodic force, large fluctuations

## Abstract

The dynamics of systems near tipping points attract considerable attention in the context of climate change, ecological regime shifts, disease spreading, and other complex systems undergoing transitions. In particular, the duration and cause of transitions between states remain subjects of ongoing debate. We address these questions by applying the large-fluctuation framework to analyse noise-induced transitions in a widely studied tipping model describing dynamics near a fold bifurcation. As complex systems are typically not in equilibrium, we include cyclic perturbations representing, for example, diurnal variations, seasonal cycles, solar activity oscillations, and Milankovitch cycles in the climate system. We investigate how the frequency and type of cyclic perturbation influence noise-induced transitions between states by examining the fluctuational force. Two types of periodic perturbations, additive and multiplicative, representing B- and R-tipping, are considered. We show, first, that depending on the type of cyclic perturbation, the fluctuations need to be synchronised with different perturbation phases to induce the transition. Secondly, we demonstrate that the transition duration depends on the perturbation frequency: when the periodic perturbation is slower than the system’s relaxation rate, the transition occurs within a single oscillatory cycle, whereas high-frequency perturbations can significantly prolong the transition time.

## 1. Introduction

Tipping points are typically associated with complex natural systems that evolve continuously under varying environmental conditions. The consideration of a tipping point is based on an analogy with the classical slip-or-tip problem [1], where the task is to estimate the minimal force, applied at a given point of a box, that causes it to tip. While the textbook problem is static, the tipping point in a natural system is a dynamical problem that focuses on the transition between states, with emphasis on the duration of this transition. For example, if the climate system approaches a tipping point, a key question is how rapidly it will exhibit distinctly new behaviour.

As in complex physical phenomena, such as phase transitions, an order parameter representing tipping dynamics is selected, for example, the global average temperature in the climate system [2], and its evolution is discussed. Often, the dynamics of the order parameter are described as a motion of a particle in a one-dimensional bistable potential [3,4,5]. Such a simplification enables efficient adaptation of existing analytical tools for the analysis of a tipping point. The adaptation leads to the widely accepted classification [5] of tipping points and links previous developments to a focus on transition features. A transition resulting from an additively and slowly changing force that leads to the loss of the initial state is called B-tipping; the letter B stands for bifurcation. A multiplicative force is associated with R-tipping; the letter R stands for rate-dependent. A transition induced by a stochastic force is called N-tipping; the letter N stands for noise-induced. The B- and R-tipping transitions are driven by monotonously changing force. In N-tipping, a slow force is implicitly present, and noise acts on a much faster timescale, inducing a transition from a stable state via an unstable one to another stable state. In all these scenarios, states are equilibrium points. Natural systems are open, and a more realistic consideration should include non-equilibrium forces. The significance of cyclic perturbations in natural phenomena is well recognised, and the timescale of the perturbation can vary in a wide range [6]. For example, in the climate system, perturbation timescales range from tides (hours) through the annual cycle and solar irradiation (decades) to Milankovich forcing (millennia) [6]. It has been shown [7,8,9] that cyclic changes modify B-tipping by slowing down or speeding up the transition depending on the timescale of the cyclic force.

However, the main feature of open systems is fluctuations, the inclusion of which provides a realistic tipping scenario. Therefore, B- and R-tipping should be considered together with N-tipping, and a cyclic (periodic) perturbation should be included. In such settings, fluctuations (noise) cause tipping, and the features of B- and R-tipping, along with the parameters of the periodic perturbation, interact with the fluctuations. This paper aims to examine this interplay. In particular, we propose analysing noise-induced tipping in the presence of a cyclic perturbation by utilising the large-fluctuation approach [10]. This approach has already demonstrated its value in analysing non-equilibrium periodically driven stochastic systems by providing a tool for studying the dynamics of transition paths [11,12,13]. Additionally, it allows us to identify the fluctuational force leading to the transition by exploring the similarity between the large-fluctuation approach and optimal control theory [14,15,16,17]. We adapt this strategy to estimate the transition duration for tipping and focus on the fluctuational force and its timing relative to the cyclic perturbation.

The structure of this paper is as follows: First, the model and the features of B- and R-tipping are introduced. Next, the strategy applied to analyse noise-induced transitions is presented. The interplay among noise, tipping types, and the parameters of cyclic perturbations is then investigated. Finally, the results and possible extensions of the presented approach are discussed in Section 5.

## 2. Model

A simplified yet powerful tipping model takes the form of a one-dimensional overdamped bistable oscillator [5]. In the presence of a cyclic perturbation and noise, it has the following form:

(1) $$\begin{array}{ccc} \dot{x} & = & -\frac{\partial U(x,t)}{\partial x}+\sqrt{2D}\xi \left(t\right)\;, \end{array}$$

(2) $$\begin{array}{ccc} U\left(x,t\right)\equiv U_{B}\left(x,t\right) & = & -\frac{x^{2}}{2}+\frac{x^{4}}{4}-s_{0}x-s\left(t\right)x\;, \end{array}$$

(3) $$\begin{array}{ccc} U\left(x,t\right)\equiv U_{R}\left(x,t\right) & = & -\frac{y^{2}}{2}+\frac{y^{4}}{4}-s_{0}x,\;\;y=x+s\left(t\right)\;, \end{array}$$

(4) $$\begin{array}{ccc} s(t) & = & A\sin(\Omega t)\;. \end{array}$$

**Figure 1:**
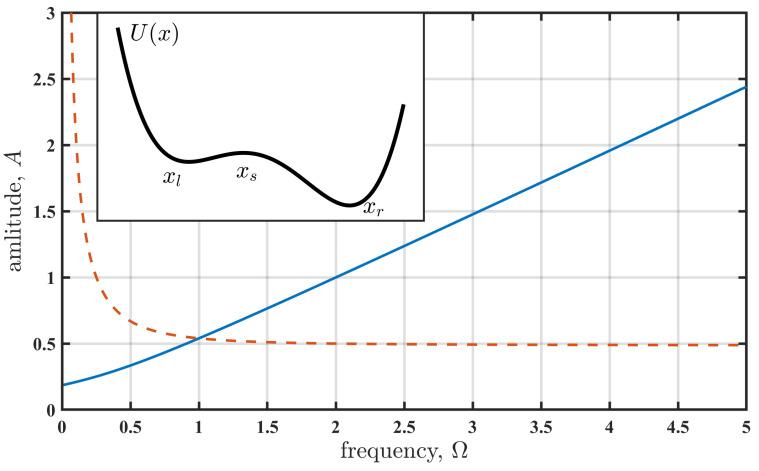
The diagram shows the parameters’ values for the fold bifurcation of cycles on the amplitude–frequency plane. The solid (blue) and dashed (red) lines correspond to the additive, Equation (2), and multiplicative, Equation (3), perturbation, respectively. Below the lines, there exist three cycles: two stable cycles associated with states $x_{l}$ and $x_{r}$ and an unstable cycle around $x_{s}$. Only one cycle associated with state $x_{r}$ exists above the line. The inset shows the shape of the potential $U\left(x\right)=-\frac{x^{2}}{2}+\frac{x^{4}}{4}-s_{0}x$.

Note that all variables and parameters are dimensionless. Here, *x* is a variable that reflects the state of the system. The last term in Equation (1) is white Gaussian noise of intensity *D*. The potential profiles $U_{B}$ and $U_{R}$ correspond to B- and R-tipping, respectively. In the latter, the cyclic perturbation is multiplicative, while it is additive in the former. The model, Equations (1)–(4), is similar to those used for tipping classification [5], but there are some differences. The periodic term, s(t), is absent in that classification. Also, for B-tipping, the parameter $s_{0}$ in Equation (2) is slowly and monotonically time-dependent; $s_{0}=at$. For R-tipping, the variable *y* in Equation (3) has the form y=x+at, and $s_{0}$ is constant. In both cases, the parameter *a* specifies the rate at which the system approaches a bifurcation. In the present consideration, we assume that *a* is small, $a\ll \frac{2\pi }{\Omega }$, and a≪τ, where τ≈1 is the relaxation time of oscillator (1). This implies that the system approaches tipping on a slower timescale than the action of the periodic perturbation s(t). The oscillator is at equilibrium when both the perturbation s(t) and noise ξ(t) are absent. The parameter $s_{0}=0.2$ is a bias term that makes the potential asymmetric (see the inset in Figure 1). We consider the transition from the vicinity of the left state $x_{l}$ to the right state $x_{r}$ via the intermediate state $x_{s}$.

The cyclic perturbation s(t), Equation (4), transforms the equilibrium states of the oscillator (1) into cycles. Acting alone, without noise (D=0), the force s(t) induces a transition from $x_{l}$ to $x_{r}$ (see the inset in Figure 1) via the fold bifurcation of cycles. Figure 1 shows a bifurcation diagram, indicating the values of amplitude *A* at the corresponding frequencies Ω where the bifurcation occurs. The diagram highlights the differences between the additive perturbation, Equation (2), and the multiplicative perturbation, Equation (3). For the additive perturbation (B-tipping), the amplitude increases monotonically with frequency. In contrast, the amplitude of the multiplicative perturbation (R-tipping) diverges as the frequency approaches zero and shows only weak dependence on frequency when it is higher than the relaxation rate of the oscillator.

## 3. Approach to Estimate the Duration of the Noise-Induced Transition and Corresponding Fluctuational Force

The large-fluctuation approach [10,18] transforms noisy dynamics into deterministic patterns by focusing on the most probable transition path. For the model (1), these patterns are described by the auxiliary system of equations in the following form [10]:

(5) $$\begin{array}{c} \begin{array}{c} \dot{x}=-\frac{\partial U(x,t)}{\partial x}+p_{x}\\ \dot{p_{x}}=\frac{\partial^{2}U\left(x,t\right)}{\partial x^{2}}p_{x} \end{array}\;, \end{array}$$

where the additional variable $p_{x}$ corresponds to the fluctuational force with a minimal action:

(6) $$\begin{array}{c} S_{m}=\int_{0}^{T}p_{x}^{2}\left(t\right)dt\;. \end{array}$$

The action appears as an exponential factor in the probability of observing a particular fluctuational trajectory, $P\left(x\left(t\right)\right)\propto \exp\left(\frac{-S_{m}}{D}\right)$. Thus, the minimal action $S_{m}$ determines the most probable path. Although the large-fluctuation approach was formally developed in the limit D→0, its applicability to real situations with finite noise is well documented [10,19,20].

The approach [18] provides a framework for analysing noise-induced transitions by reformulating the stochastic problem, Equation (1), as a deterministic one, Equation (5). Within this framework, fluctuations drive the system’s trajectory from a stable state to an unstable state that lies at the boundary between two stable states. After reaching this boundary state, the system relaxes to another stable state with zero fluctuation-induced action. Consequently, the most probable trajectory connects a stable and an unstable state in system (1). Since these states are cycles, system (5) is converted into a discrete-time map by considering the system’s solution at specific time instances, $t^{n}=\frac{2\pi }{\Omega }n$. The solution, x(t) and $p_{x}\left(t\right)$, of system (5) is obtained numerically, and the corresponding discrete-time sequence $x\left(t^{n}\right),p_{x}\left(t^{n}\right)\equiv x^{n},p_{x}^{n}$ is formed. This procedure formally leads to a map of the following form:

(7) $$\begin{array}{c} \begin{array}{c} x^{n+1}=F\left(x^{n},p_{x}^{n}\right)\\ p_{x}^{n+1}=G\left(x^{n},p_{x}^{n}\right) \end{array}\;. \end{array}$$

Cycles of system (5) correspond to fixed points in the map (7). Moreover, stable and unstable cycles of oscillator (1) are converted into saddle cycles of system (5), which correspond to saddle fixed points in map (7). Since the map is two-dimensional, each saddle point possesses a one-dimensional stable manifold and a one-dimensional unstable manifold.

**Figure 2:**
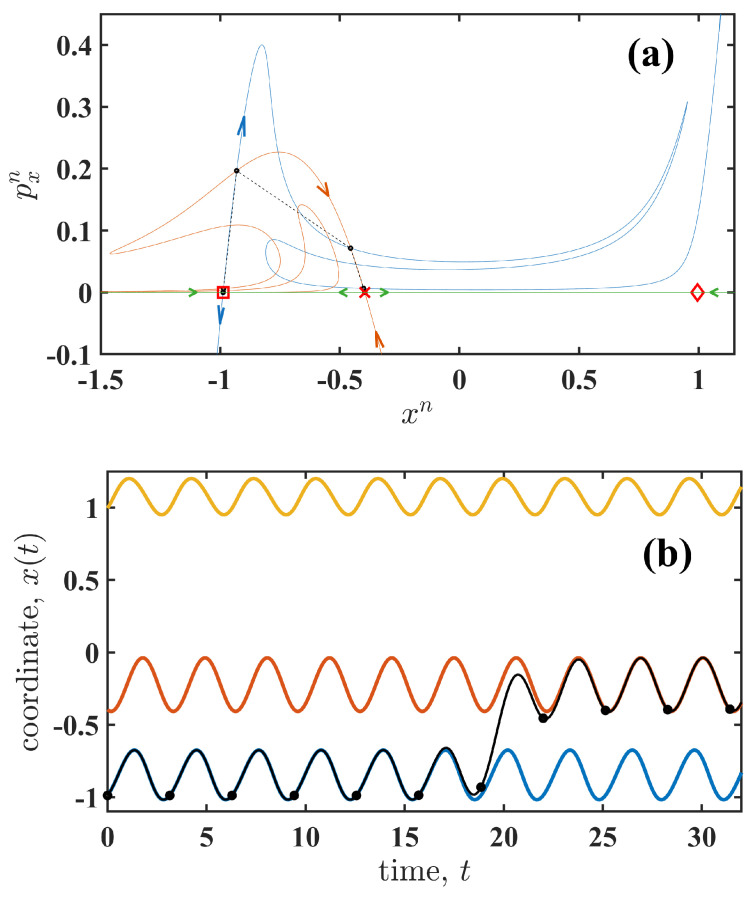
(**a**) shows the state space of map (7) for Ω=2.0 and A=0.95. Fixed points corresponding to cycles around the stable states $x_{l}$ and $x_{r}$ and the unstable state $x_{s}$ are shown by red markers □, ⋄, and ×, respectively. The line $p_{x}^{n}=0$ is shown in green. Unstable and stable manifolds, extending from line $p_{x}^{n}=0$, are shown by blue and red thin lines, respectively. Note that only a portion of the manifolds is displayed to simplify the figure. Arrows indicate the direction of trajectory evolution. Black dots correspond to the heteroclinic trajectory with the minimal action, i.e., the most probable path. Black dashed lines illustrate the path’s dynamics. (**b**) shows the most probable path in the continuous-time system (5) by a black line. The black dots correspond to those in (**a**). In (**b**), stable cycles are shown by blue and yellow lines and the unstable cycle is shown in red.

Figure 2a shows the state space of the map (7). Note that the equivalent state space of the system (1) corresponds to the line $p_{x}^{n}=0$, where the fixed points (cycles) are located. These fixed points divide the line into several regions, within which the system (1) evolves according to the green arrows (see Figure 2a). The unstable cycle around state $x_{s}$ forms a boundary between the two stable cycles around states $x_{l}$ and $x_{r}$. Trajectories corresponding to the unstable and two stable cycles are shown in Figure 2b.

In the extended map (7), each fixed point has manifolds oriented in the opposite direction to those on the line $p_{x}^{n}=0$ (see Figure 2a). Specifically, a fixed point corresponding to a stable cycle of (1) has a stable manifold along the line $p_{x}^{n}=0$ and an unstable manifold (blue thin line in Figure 2a) extending away from the line and the fixed point. Conversely, for a fixed point corresponding to an unstable cycle of (1), the manifold along $p_{x}^{n}=0$ is unstable, while the stable manifold (red thin line in Figure 2a) directs trajectories toward the fixed point. Thus, each fixed point corresponding to a cycle of system (1) is a saddle.

In a small vicinity of the saddle points, the manifolds follow the eigenvectors of the Jacobian of the fixed points of map (7). The Jacobian can be evaluated numerically using finite differences. For example, the Jacobian terms $\partial F/\partial x^{n}$ and $\partial G/\partial x^{n}$ are estimated using the following expressions:

(8) $$\begin{array}{c} \begin{array}{c} \frac{\partial F}{\partial x^{n}}=\frac{x^{1}-x^{0*}}{h_{x}}\\ \frac{\partial G}{\partial x^{n}}=\frac{p_{x}^{1}-p_{x}^{0*}}{h_{x}} \end{array}\;, \end{array}$$

where $\left(x^{0*},p_{x}^{0*}\right)$ are the coordinates of the fixed point, and $\left(x^{1},p_{x}^{1}\right)$ is the solution of the map (7) obtained for the initial condition $\left(x^{0*}+h_{x},p_{x}^{0*}\right)$. Similarly, the initial condition $\left(x^{0*},p_{x}^{0*}+h_{x}\right)$ is used to evaluate the Jacobian terms $\partial F/\partial p_{x}^{n}$ and $\partial G/\partial p_{x}^{n}$. By considering a set of initial conditions along the eigenvectors of the Jacobian, a manifold of saddle points can be estimated.

The most probable path corresponds to a heteroclinic trajectory (black circles in Figure 2a), which is the intersection of two manifolds and has minimal action $S_{m}$. One manifold is unstable (blue thin line in Figure 2a) and originates from a saddle point associated with a stable cycle (marker □). The other manifold is stable (red thin line in Figure 2a) and corresponds to a saddle point associated with an unstable cycle (marker ×). Thus, the most probable path is the solution of a boundary value problem with boundaries defined by the saddles’ manifolds. The action-plot method [21] can then be applied to find the solution of this boundary value problem with minimal action.

**Figure 3:**
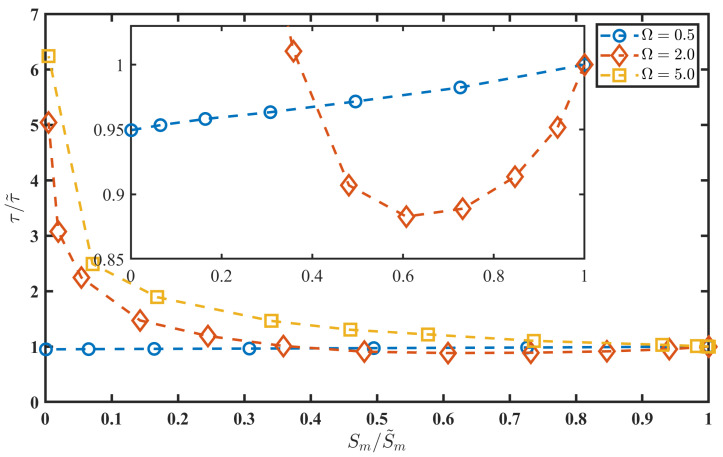
The dependences of the normalised duration $\tau /\tilde{\tau }$ on the normalised action $S_{m}/{\tilde{S}}_{m}$ for different frequency values: Ω=0.5 (marker ∘, blue line), Ω=2.0 (marker ⋄, red line), and Ω=5.0 (marker □, orange line). The dependences are the same for the additive (2) and multiplicative (3) perturbations. The inset shows a zoomed-in part of the dependences for Ω=0.5 (marker ∘, blue line) and Ω=2.0 (marker ⋄, red line).

Since the map (7) is estimated numerically using system (5), the continuous-time trajectories are also calculated. An example of a continuous-time trajectory x(t) of system (5) is shown in Figure 2b by the black line. The black points indicate the corresponding trajectory of the map (7). Therefore, the solution of the boundary value problem for map (7) provides the trajectories of x(t) and $p_{x}\left(t\right)$, corresponding to the most probable path and the fluctuational force, respectively. The trajectories are obtained over the time interval $t\in [t_{i},t_{f}]$, where $t_{i}$ corresponds to the boundary condition on the unstable manifold and $t_{f}$ corresponds to the boundary condition on the stable manifold. The interval duration, $T=t_{f}-t_{i}$, varies with the system’s parameters. For simplicity, we set $t_{i}=0$. The action is then defined by expression (6). Formally, a heteroclinic trajectory is defined over an infinite interval, i.e., $t_{i}\to -\infty$ and $t_{f}\to \infty$. Using a finite interval *T* introduces an error in estimating $S_{m}$. However, this error is comparable to that arising from the numerical methods used to solve differential Equation (5), since the boundary conditions are selected within a small vicinity of the saddle points. The Runge–Kutta fourth-order method [22] with a step size h<0.001 was used for the numerical solution of (5). To estimate the transition duration, the time-dependent action

(9) $$\begin{array}{c} S\left(t\right)=\int_{0}^{t}p_{x}^{2}\left(t^{\prime }\right)dt^{\prime }\;,\;t\in \left[0,T\right]\; \end{array}$$

calculated along the most probable path was considered. Expression (9) defines a monotonic function with a maximum value $S_{m}=S\left(T\right)$, as given by expression (6). The action duration, τ, is defined as the time interval during which the following two conditions are satisfied: $S\left(t\right)>0.005\;S_{m}$ and $S\left(t\right)<0.995\;S_{m}$. Note that this approach is also applicable to a non-perturbed oscillator, when s(t) is absent (A=0). Quantities estimated for the non-perturbed oscillator are denoted with a tilde. In this case, the duration and action are $\tilde{\tau }=5.797$ and $\tilde{S}={\tilde{S}}_{m}=0.327$, respectively, when A=0. We used these quantities for the normalisation in Figure 3.

## 4. Results

The value $\tilde{\tau }$ specifies the timescale of the noise-induced transition in the non-perturbed system (A=0). Accordingly, a cyclic perturbation with a period longer than $\tilde{\tau }$ can be considered a slowperturbation, while it is fast in the opposite case. We select the frequency Ω=0.5 in (4) for the slow-perturbation case, and the frequencies Ω=2.0 and Ω=5.0 for the fast-perturbation case. For each frequency, the amplitude *A* is varied from zero up to the value corresponding to the fold bifurcation (see Figure 1), and the noise-induced transition durations τ and actions $S_{m}$ are estimated using the boundary value problem solution of system (5). These quantities are evaluated for both additive (2) and multiplicative (3) perturbation types, corresponding to B- and R-tipping [5]. An interesting observation reported in Figure 3 is the identical relationship between the normalised duration and the normalised action for the additive (2) and multiplicative (3) perturbations. Note that the additive and multiplicative perturbations yield the same normalised action $S_{m}/{\tilde{S}}_{m}$ for different values of amplitude *A*. However, the perturbation frequency strongly affects these relationships. For slow perturbations, the transition duration is not significantly affected, decreasing slightly as the action decreases (i.e., as amplitude *A* increases). In contrast, for fast perturbations, the duration increases by several times for large amplitudes *A* (small $S_{m}/{\tilde{S}}_{m}$), indicating a substantial slowdown of the tipping transition.

The transition slowdown follows the power law

(10) $$\begin{array}{c} \frac{\tau }{\tilde{\tau }}=C{\left(\frac{S_{m}}{{\tilde{S}}_{m}}\right)}^{\gamma }\;, \end{array}$$

where γ≈0.34 and the constant *C* depends on the frequency Ω. For Ω=5.0, the entire dependence follows the power-law scaling. For Ω=2.0, the dependence is more complex. Initially, the cyclic perturbation at Ω=2.0 slightly speeds up the transition (see the inset in Figure 3). Subsequently, the transition duration increases, and the duration–action relationship follows the power law (10) for normalised actions close to zero.

**Figure 4:**
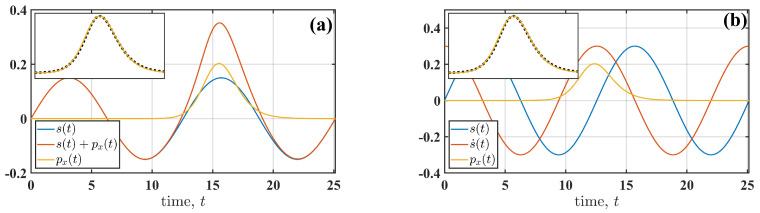
An illustration of cooperation between the fluctuational force $p_{x}\left(t\right)$ and cyclic perturbation s(t) to induce the transition between states for additive (**a**) and multiplicative (**b**) perturbations. Perturbation s(t) and force $p_{x}\left(t\right)$ are shown with blue and orange solid lines, respectively. The red solid line denotes the sum $s\left(t\right)+p_{x}\left(t\right)$ and $\dot{s}\left(t\right)$ in (**a**) and (**b**), respectively. In both figures, the inset shows the shapes of the fluctuational forces obtained by normalising the forces by their maximal values: $p_{x}\left(t\right)/p_{x}^{m}$. In the inset, the black dotted line corresponds to the fluctuational force of the non-perturbed oscillator (A=0). The normalised action is $\frac{S_{m}}{{\tilde{S}}_{m}}\approx 0.3$ for both figures.

Further insight into the observed dependencies (Figure 3) is obtained by examining the fluctuational force $p_{x}\left(t\right)$ for different parameters of the perturbation (4) and for the perturbation types (2) and (3). We begin with the slow perturbation, Ω=0.5. Figure 4 illustrates the similarities and differences between additive (Figure 4a) and multiplicative (Figure 4b) perturbations. The force shapes (see the insets in Figure 4a,b) are similar to those of the non-perturbed system (A=0). However, the timing of $p_{x}\left(t\right)$ relative to the cyclic perturbation s(t) is distinct. For the additive perturbation (Figure 4a), the fluctuation synchronises with s(t), which increases the amplitude of s(t) required to induce the transition. In contrast, for the multiplicative perturbation (Figure 4b), the fluctuation synchronises with the rate of change of s(t) ($\dot{s}\left(t\right)=\frac{ds(t)}{dt}$). This behaviour highlights a signature of R-tipping [5], where the transition is induced by changes in the perturbation rate. Thus, the phase of s(t) at which the fluctuational force acts depends on the type of perturbation (additive or multiplicative), and this observation holds for all frequencies of the cyclic perturbation s(t).

**Figure 5:**
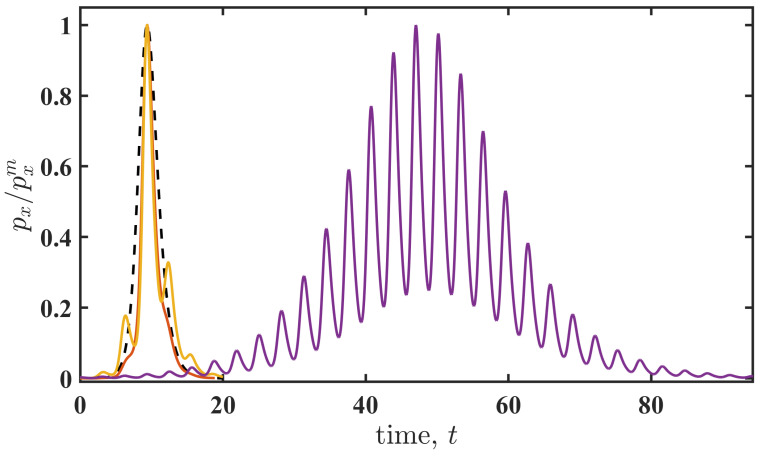
The shapes of the fluctuational force for different values of the normalised action for Ω=2.0. The shapes are obtained by the normalisation of the force: $p_{x}\left(t\right)/p_{x}^{m}$. The black dashed line corresponds to $S_{m}/{\tilde{S}}_{m}=1$, the red solid line corresponds to $S_{m}/{\tilde{S}}_{m}\approx 0.6$, the orange solid line corresponds to $S_{m}/{\tilde{S}}_{m}\approx 0.36$, and the violet solid line corresponds to $S_{m}/{\tilde{S}}_{m}\approx 0.002$.

While for the slow perturbation (Ω=0.5), the shape of $p_{x}\left(t\right)$ is close to that of the non-perturbed system (A=0), fast perturbations lead to adjustments in the force’s shape (Figure 5). The shape envelope tends to follow that of the non-perturbed system (black dashed line in Figure 5), but there is a pronounced cyclic modulation at the perturbation frequency Ω. As the perturbation amplitude *A* increases, corresponding to a decrease in the normalised action $S_{m}/{\tilde{S}}_{m}$, the envelope widens and the modulation depth increases. These changes in the fluctuational force $p_{x}\left(t\right)$ result in a prolonged transition for large amplitudes *A*.

**Figure 6:**
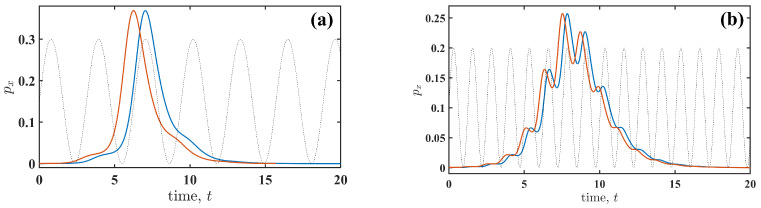
The fluctuational force $p_{x}\left(t\right)$ is shown for (**a**) Ω=0.2 and $S_{m}/{\tilde{S}}_{m}\approx 0.6$ and (**b**) Ω=0.5 and $S_{m}/{\tilde{S}}_{m}\approx 0.46$. The blue and red solid lines correspond to additive (2) and multiplicative (3) perturbations, respectively. The black dashed line shows perturbation s(t) with an amplitude value adjusted for visualisation. Note that the amplitude *A* is different for additive and multiplicative types of perturbation s(t).

The changes in the shape of $p_{x}\left(t\right)$ are similar for both additive (2) and multiplicative (3) perturbations (Figure 6). Figure 6a shows the fluctuational force $p_{x}\left(t\right)$ for both perturbation types when the transition duration is shorter than that of the non-perturbed system. The shape corresponding to a prolonged transition duration is shown in Figure 6b. It is evident that the identical shapes are shifted relative to each other in order to synchronise with different phases of s(t), depending on the perturbation type.

**Figure 7:**
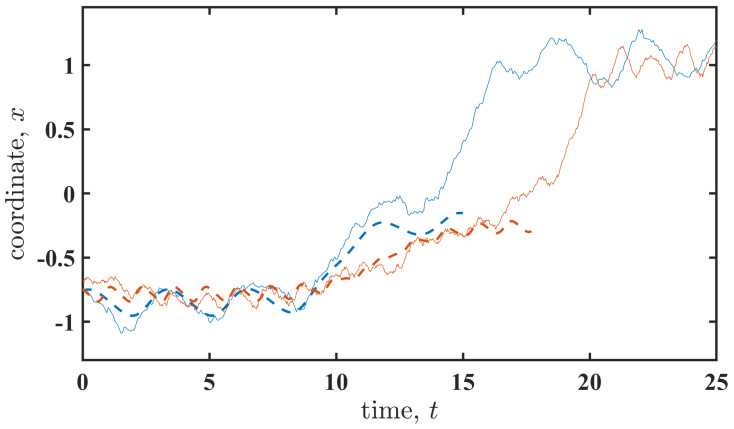
The transition paths (solid lines) obtained by solving the stochastic differential Equation (1) and the corresponding most probable paths (dashed lines) obtained using the large-fluctuation approach are shown for multiplicative perturbation (3) and the following parameters: Ω=2.0 and A=0.2 ($S_{m}/{\tilde{S}}_{m}\approx 0.6$), indicated by the blue colour, and Ω=5.0 and A=0.3 ($S_{m}/{\tilde{S}}_{m}\approx 0.46$) denoted by the red colour.

The results presented above were obtained using the large-fluctuation approach, in which stochastic (noisy) dynamics are represented by corresponding deterministic patterns in the limit of small noise intensity, D→0. To verify the relevance of these results to the original stochastic system (1), we simulate the system and collect the transition paths. Figure 7 shows examples of transition paths (solid lines) obtained by solving the stochastic differential Equation (1) alongside the corresponding most probable paths (dashed lines) obtained using the large-fluctuation approach. The latter provides trajectories that terminate at an unstable state. As observed, the stochastic trajectories closely follow the most probable paths. After reaching the unstable state, the stochastic trajectories follow relaxation paths that require no fluctuational action. Note that the relaxation path also depends on the cyclic perturbation s(t), and this dependence can be further clarified by considering the non-stochastic (D=0) system. While such an analysis is beyond the scope of this paper, we expect that the duration of the relaxation path depends on the perturbation properties in a manner similar to that of the activation path.

## 5. Conclusions

We have shown that the large-fluctuation approach can comprehensively describe noise-induced dynamics near a tipping point. This approach is applicable to non-equilibrium situations in which the system is subjected to cyclic perturbations. It allows us to estimate the duration of noise-induced transitions and to characterise their features through the fluctuational force. Our analysis highlights both the similarities and differences between the two types of cyclic perturbations: additive (2) and multiplicative (3). The results indicate that the transition duration τ does not depend on the type of perturbation s(t). Instead, τ is determined by the normalised action $S_{m}/{\tilde{S}}_{m}$, that is, by the perturbation amplitude *A*, and by the perturbation frequency Ω. However, the perturbation type dictates the timing of the fluctuational force $p_{x}\left(t\right)$. For additive perturbations, $p_{x}\left(t\right)$ synchronises with s(t), whereas for multiplicative perturbations, $p_{x}\left(t\right)$ synchronises with the perturbation rate $\dot{s}\left(t\right)$. For both types of perturbations, the relationship between the period of s(t) and the transition duration $\tilde{\tau }$ of the non-perturbed system (A=0) determines the shape of the fluctuational force $p_{x}\left(t\right)$ and the transition duration τ. When the cyclic perturbation is slow, both $p_{x}\left(t\right)$ and τ vary only slightly with the perturbation amplitude *A*. In contrast, for fast perturbations, the shape of $p_{x}\left(t\right)$ is modulated at the perturbation frequency Ω, and the modulation depth increases with amplitude *A*. These changes in the fluctuational force $p_{x}\left(t\right)$ are accompanied by a longer transition duration. For fast perturbations, the transition duration τ depends on the normalised action according to a power-law relationship, so that τ can increase by an order of magnitude at large perturbation amplitudes *A*. This increase occurs when the barrier between states is small but finite, requiring fluctuations to induce the transition. In the context of tipping phenomena, this result implies a pronounced slowdown of the transition. For instance, in the climate system, including cyclic perturbations can help explain the absence of dramatic climatic changes when the global average temperature is close to a tipping point, even when fluctuations are sufficient to trigger transitions [2].

The presented approach can be extended in several directions. One direction is the control of noise-induced transitions, for example, by preventing them with a control force [17]. Another direction is the application of the approach to more complex tipping models, although this may face computational challenges when estimating heteroclinic trajectories in high-dimensional systems. A further possible extension is to incorporate the effects of strong noise. In the current analysis, we assume that both the cyclic perturbation and the noise amplitude remain constant over time and are decoupled from the slowly varying parameter $s_{0}=at$. In this scenario, the system has sufficient time to reach a quasi-stationary distribution around a stable state, making the large-fluctuation approach applicable. However, if the change in $s_{0}=at$ becomes comparable to the period of the cyclic perturbation, or if the properties of the perturbation or noise amplitude change rapidly in time, the present approach is no longer valid. In such cases, the method could be extended using the framework described in works [23,24].

## Data Availability

The original contributions presented in this study are included in the article. Further inquiries can be directed to the corresponding author(s).
